# Olfactory Dysfunction: A Clinical Marker of COVID-19

**DOI:** 10.31729/jnma.5658

**Published:** 2021-01-31

**Authors:** Apar Pokharel

**Affiliations:** 1 College of Medical Sciences, Chitwan, Nepal

**Keywords:** *anosmia*, *olfactory dysfunction*, *SARS-CoV-2*

## Abstract

Due to the rapid spread of the SARS-CoV-2 virus, many health systems worldwide are overwhelmed, leading to the triggering of the scarcity of medical resources. The identification of indicators that require hospital admission help in the efficient allocation of medical resources. Olfactory impairment is also one of the indicators of COVID-19 infection. Many studies have analyzed olfactory dysfunction in COVID-19 with a variable prevalence rate but underreporting of this problem is very much likely as the problem is considered benign. Many scientific societies have stated that olfactory dysfunction is a frequent symptom of COVID-19 and have published recommendations for it.

## INTRODUCTION

The COVID-19 is an ongoing viral pandemic that started in December 2019 from Wuhan, Hubei province, China, and quickly spread to the rest of the world.^1^ World Health Organisation (WHO) named the disease COVID-19 on February 12, 2020. As of 10th September, 2020, WHO reports a total number of 27.9 million people have been diagnosed with COVID-19 infection, with 905 thousand deaths involving 214 countries and territories.^2^ COVID-19 disease is responsible for the longest pandemic since the 1918 H1N1 influenza outbreak.^3^ Human-to-human transmission is occurring at an exponential rate, which has led to steep curves in the incidence of disease in many countries.^4^

According to clinical studies, fever, cough, dyspnea, sputum production, arthralgia, myalgia, diarrhea, headache, rhinorrhea, and sore throat are the most commonly reported clinical features.^5,6^ Among these, fever and cough with lymphocytopenia and ground-glass opacity changes on chest computed tomography the main manifestations of COVID-19 patients.^1^ In severe infection, acute cerebrovascular diseases, skeletal muscle injury, and impaired consciousness have also been reported.^7^

However, recent studies have shown olfactory and gustatory dysfunctions as newer clinical features. People with loss of smell and taste are three times more likely to have contracted the virus if they have other symptoms described by the World Health Organisation.^8^ Post viral olfactory dysfunction is a common phenomenon that occurs due to inflammatory reactions to the nasal mucosa and is accompanied by rhinorrhoea. However, olfactory dysfunction in COVID-19 can occur even without rhinorrhoea.^9,10^ The severity and prevalence of olfactory dysfunction among COVID-19 patients still remain unclear. We have searched databases like PubMed, Cochrane Clinical Trials, ScienceDirect, Lilacs, Scopus, and Google Scholar. This review summarizes various studies’ findings on olfactory dysfunction during the COVID-19 pandemic, its pathophysiology, and strategies to cope with this problem.

## PATHOPHYSIOLOGY

Post-viral upper respiratory tract infections (8-45%), nasal sinus disease (7-56%), head trauma (8-20%), toxins/drugs (2-6%), and congenital loss (0-4%) are the leading causes of olfactory dysfunction seen in clinics of USA, Europe, and Japan.^11^ Viral upper respiratory tract infections are responsible for the cause of postviral anosmia.^12,13^ Post-viral anosmia is more common in female and middle-aged and older individuals.^14^ Such anosmia has a favorable prognosis.^12^ Suzuki et al. found coronavirus, rhinovirus, parainfluenza virus, and Epstein-Barr virus in patients’ nasal secretions suffering from post-viral olfactory disorders.^9^ Different kinds of viruses, including coronavirus as HCoV-229E, can cause post-viral olfactory loss. Post viral olfactory loss is usually of long duration, with 80% subjective recovery after one year of follow-up.^15^ In COVID-19, the anosmia is acute, lasting for one week.^16–18^

Seven different strains of coronavirus can cause human infection, namely Severe Acute Respiratory Syndrome Corona Virus-2 (SARS-CoV-2), Severe Acute Respiratory Syndrome Corona Virus (SARS-CoV), Middle East Respiratory Syndrome Corona Virus (MERS-CoV), HCoV-NL63, HCoV-229E, HCoV-HKU1, and HCoV-OC43.^19^ SARS-CoV-2 is a 29,903 bp singlestranded RNA coronavirus.^20^ Both SARS-CoV and SARS-CoV-2 belong to the coronavirus family's betagenus and share an 82% similar genetic sequence.^21^ On studying the pathology of coronavirus infection using the rhesus macaques model, it was revealed that the primary pathogenic site for the SARS-CoV-2 virus was the nose and throat. The viral load in the patient's nasal cavity was higher than the pharynx.^22^

ACE-2 receptor inhibitor in the nasal mucosa participates in respiratory inflammatory diseases by regulating the level of inflammatory peptides like bradykinin.^23^ However, in COVID-19 patients, alteration of the sensation of smell is not accompanied by rhinitis and other inflammatory components.^18,24^ So, one hypothesis is that the transmission could damage the olfactory pathway by the virus. In 2006, Hwang et al. described a case of anosmia that persisted for two years post-SARS infection.^25^ In 2001, Schwob et al. and Youngentob et al., on examining the olfactory bulb of SARS-CoV patients, found an abnormal predominance of immature neurons, indicating accelerated turnover of cells.^26,27^ This reduction in neurons’ lifespan is due to the loss of trophic support supplied by the olfactory bulb to the sensory neurons.^26,27^ In 2008, Netland et al. hypothesized that neuronal death in SARS-CoV infection occurs due to the production of interleukin-6 (IL-6) under the stimulation of viral N-spike protein.^28^ However, various COVID-19 case series report a high recovery rate of olfactory function within two weeks of infection.^18,29,30^ In addition to this, the frequency of central nervous system symptoms is much lower than SARS-CoV infection. This suggests that the target of the COVID-19 virus may not be neurons but other supporting cells that express ACE-2 receptors like sustentacular cells, microvillar cells, Bowman's gland cells, horizontal basal cells, and olfactory bulb pericytes.^31^

COVID-19, like SARS-CoV, infects cells through interactions between its spike (S) protein and ACE-2 protein on target cells. ACE-2 binding affinity of COVID-19 spike protein ectodomain is 10-20 fold higher than SARS-CoV spike protein.^20^ This interaction requires cleavage of S-protein by cell surface protease TMPRSS2. On previously published data of bulk RNA-Seq data derived from the whole olfactory epithelium of humans, no genes for ACE-2 nor TMPRSS2 were detected in the mature olfactory sensory neuroepithelium. However, ACE-2 was seen in the sustentacular cells and horizontal basal cells. This shows that sustentacular cells and horizontal basal cells are potential targets of the COVID-19 virus. Sustentacular cells structurally support sensory neurons, phagocytose and/or detoxify potentially damaging agents and maintain local salt and water balance. Horizontal basal cells act as reserve stem cells, which get activated upon tissue damage.^31^

The SARS-CoV-2 virus can cause acute onset anosmia by damaging the olfactory epithelium.^12^ A possible mechanism is that viruses can use the olfactory nerve to enter into the central nervous system like influenza A virus, herpes virus, poliovirus, rabies virus, parainfluenza virus, adenovirus, and Japanese encephalitis virus.^10^ SARS-CoV virus showed transneuronal penetration through the olfactory bulb in mouse models resulting in the rapid intracranial spread.^32^

## LITERATURE REVIEW

Studies conducted in Spain, Italy, United Kingdom, Belgium, France, the United States, and Iran showed the prevalence of olfactory dysfunction in the range of 33.9 to 68%. The studies were conducted through non-contact methods like online questionnaires and telephone interviews. However, an olfactory function test (OFT) was not done in these cross-sectional studies.^8,29,33–35^ OFT was used in a study done by Ottaviano et al., who reported hyposmia as a primary symptom in six COVID-19 patients. The six odor smell test confirmed hyposmia.^36^ Moein et al. used 40 odorant University of Pennsylvania Smell Identification Test. It concluded that 98% of COVID-19 subjects had some olfactory dysfunction, and only 35% of them were aware of this dysfunction before testing.^3^ A study done by the Mayo clinic using artificial intelligence showed anosmia as one of the features of COVID-19 patients. It also showed that the prevalence of anosmia was 28.6 fold higher than in COVD-19 positive patients.^37^

Anosmia is one of the common symptoms of SARS-CoV-2 infection.^38^ Klopfenstein et al. reported that out of 114 cases of COVID-19, 54 (47%) presented with anosmia. Anosmia was seen 4.4 days after the onset of infection and lasted for 8.96 days. 98% of patients recovered within 28 days of infection.^17^ Luers et al. reported that anosmia was seen on the fourth day after the first symptom onset.^39^ Beltran-Corbellini et al. and Lee et al. reported that the duration of smell disorder lasted for seven days. They also reported that 67.7% of patients reported acute onset of anosmia.^16,40^ Moein et al. reported that only 35% of the patients were aware of olfactory dysfunction before their smell examination was done.^3^ Yan et al. found that olfactory dysfunction was ten times more common in COVID-19 positive cases than in COVID-19 negative controls. Both cases and controls have similar influenza-like symptoms.^29^ Yan et al. showed a temporal relationship of olfactory dysfunction with a resolution of overall clinical illness in the ambulatory population.^29^ Corbellini et al. suggested new-onset olfactory dysfunction is more frequent amongst COVID-19 than in other viral upper respiratory tract infections.^40^

Moein et al. reported fewer smokers in the COVID-19 cohort than in the control population.^3^ Similar findings were reported in studies done on Chinese and US people.^41,42^ It is hypothesized that smoking upregulates the expression of ACE-2 in upper airways, increasing the risk of coronavirus infection but paradoxically protects the host from acute lung injury.^43^ Smokers are less susceptible to olfactory dysfunction from industrial exposures to acrylate and methacrylate.^44^ Smoking also protect olfactory loss to some degree in Parkinson's disease.^45^ Further research is warranted to find the cause of the low frequency of involvement of COVID-19 positive smokers complaining of olfactory loss.

In a study done in the ambulatory population, patients with influenza-like symptoms and anosmia are 6 to 10 times more likely to test positive for COVID-19 infection.^29,46^ 59-86% of COVID-19 patients presented with self-reported olfactory loss.^18,29,46^ Moein et al. reported high olfactory dysfunction rates (98%) on quantitative analysis of COVID-19 patients with only 35% self-reported olfactory loss.^3^ This discrepancy between quantitative and self-reported olfactory dysfunction might be due to the lack of awareness or hyposmia under-reporting.^47^ Another reason for decreased reporting might be more severe symptoms like respiratory distress among these patients.^48^ Some studies state that patients who self-reported their olfactory loss usually have a milder form of the disease. Self-reported olfactory loss can be used as one indicator to risk-stratify patients for early determination and intervention among COVID-19 patients.^48^

A high incidence rate of olfactory dysfunction is reported in European and American countries compared to the Chinese population.^17,49^ Mao et al. reported an incidence of olfactory dysfunction to be around 5.1% in the COVID-19 positive Chinese population.^7^ Several reasons might be the cause of this difference. First is the mutation of the SARS-CoV-2 virus resulting in different genotypes. The A and C genotypes of the SARS-CoV-2 virus are more commonly seen among

Europeans and Americans, and the B genotype is more commonly seen in the East Asian population.^50^ The high pathogenicity of A and C strain for nasal cavity epithelium might cause a high prevalence of the olfactory disorder. The second reason might be that susceptibility differences among different human races might cause a difference in the prevalence of olfactory dysfunction among different races. A third reason is that people were more concerned about the primary life-threatening conditions during the outbreak in China, and the olfactory symptoms were overlooked and, therefore, under-reported. These are assumptions, and future research is warranted on this topic.^51^

An internet-based tool called Google Trends (GT) showed an abnormal elevation of anosmia topic search. Compared to the first week of March 2020, the search for anosmia topic had increased by 100% by the last week of March 2020. At present, by the second week of September 2020, the “Covid Anosmia” topic is around 33% more searched compared to the first week of March.^52^ Gane et al. revealed a strong correlation between daily research volumes in Covid anosmia topic and the number of COVID-19 patients.^53^

## PERSPECTIVES FROM OTOLARYNGOLOGY

Prof. Claire Hopkins did the first official emphasis on anosmia as an early symptom of COVID-19 infection on March 21, 2020.^54^ American academy of otolaryngology-head and neck surgery also released a statement mentioning that anosmia with dysgeusia can be seen in COVID-19 patients.^55^ American Academy of Otolaryngology-Head and Neck Surgery (AAO-HNS) also established the COVID-19 Anosmia Reporting Tool for Clinicians to allow healthcare providers to report olfactory dysfunction related to COVID-19 cases systematically.^55^ The increased number of cases with olfactory dysfunction in COVID-19 patients prompted organizations like the American Academy of Otolaryngology-Head and Neck Surgery and ENT UK to recommend the inclusion of sudden-onset loss of smell and/or taste as part of the diagnostic criteria for COVID-19 disease, as has now been done by the CDC.^54–56^

These organizations recommend that recent-onset of olfactory dysfunction alone is a sufficient symptom for self-isolation. Medical staff must use personal protective equipment (PPE) while evaluating patients with this clinical problem. Infected respiratory droplets from COVID-19 patients can get transmitted through the nose and mouth. Contact transmission by hands can also occur through virus deposited surfaces.^57^ Researchers have also suggested that the SARS-CoV-2 virus can transmit via aerosols.^58^ During this pandemic period, routine appointments must be delayed or postponed to decrease the chance of virus infection of patients or healthcare professionals. Except for emergency conditions, online telemedicine is the best option for the reduction of COVID-19 cross-infection. ^59^

## FUTURE DIRECTION

Three areas of future research are there. First, clinical data on a large cohort of the population is needed to find the frequency and recovery time of anosmia in COVID-19 infection. Second, any correlation between the nasopharyngeal viral load and the severity of olfactory dysfunction must also be investigated. Lastly, histopathological analysis of the olfactory system can also be done in patients who died of COVID-19 infection.

## CONCLUSIONS

Chemosensitive disorders like loss of olfaction is a frequent symptom of COVID-19 patients. The identification of paucisymptomatic patients is vital to manage the COVID-19 health crisis. In many centers, patients present with anosmia and ageusia with fever (>37.5) as onset symptoms are detected. Early identification of such conditions can break the chain of transmission. During the COVID-19 pandemic, attention has been given to the infected patients and frontline responders, with little attention being given to the hidden iceberg of suspected COVID-19 cases that are not yet in hospital. As widespread population testing is not possible in many countries, it is of utmost importance to identify all symptoms of COVID-19 so that a suspected case can self-isolate himself and prevent the spreading of disease. Anosmia could be used as a screening tool to identify potential cases that could be instructed to self-isolate.
